# The emerging epidemic of inflammatory bowel disease in Asia and Iran by 2035: A modeling study

**DOI:** 10.1186/s12876-021-01745-1

**Published:** 2021-05-06

**Authors:** Meysam Olfatifar, Mohammad Reza Zali, Mohamad Amin Pourhoseingholi, Hedieh Balaii, Shaghayegh Baradaran Ghavami, Maria Ivanchuk, Pavlo Ivanchuk, Saeed hashemi Nazari, Shabnam shahrokh, Siamak Sabour, Soheila Khodakarim, Hamid Asadzadeh Aghdaei, Pejman Rohani, Gholamhossein Mehralian

**Affiliations:** 1 Gastroenterology and Liver Diseases Research Center, Research Institute for Gastroenterology and Liver Diseases, Shahid Beheshti University of Medical Sciences, Tehran, Iran; 2 Basic and Molecular Epidemiology of Gastrointestinal Disorders Research Centre, Research Institute for Gastroenterology and Liver Diseases, Shahid Beheshti University of Medical Sciences, Tehran, Iran; 3 Biological Physics and Medical Informatics Department, Bukovinian State Medical University, Chernivtsi, Ukraine; 4 Internal Medicine, Physical Rehabilitation, Sports Medicine and Physical Training Department, Bukovinian State Medical University, Chernivtsi, Ukraine; 5 Prevention of Cardiovascular Disease Research Centre, Department of Epidemiology, School of Public Health and Safety, Shahid Beheshti University of Medical Sciences, Tehran, Iran; 6 Department of Clinical Epidemiology, School of Health and Safety, Shahid Beheshti University of Medical Sciences, Tehran, Iran; 7 Safety Promotions and Injury, Prevention Research Centre, Shahid Beheshti University of Medical Sciences, Tehran, Iran; 8 Department of Biostatistics, School of Medicine, Shiraz University of Medical Sciences, Shiraz, Iran; 9 Pediatric Gastroenterology, Hepatology and Nutrition Research Center, Research Institute for Children Health, Shahid Beheshti University of Medical Sciences, Tehran, Iran; 10 School of Pharmacy, Shahid Beheshti University of Medical Sciences, Tehran, Iran

**Keywords:** Asia regions, Iran, Inflammatory Bowel Disease, Projection, Emergency action

## Abstract

**Background:**

The projection studies are imperative to satisfy demands for health care systems and proper response to the public health problems such as inflammatory bowel disease (IBD).

**Methods:**

To accomplish this, we established an illness-death model based on available data to project the future prevalence of IBD in Asia, Iran in particular, separately from 2017 to 2035. We applied two deterministic and stochastic approaches.

**Results:**

In 2035, as compared to 2020, we expected a 2.5-fold rise in prevalence for Iran with 69 thousand cases, a 2.3-fold increment for North Africa and the Middle East with 220 thousand cases, quadrupling of the prevalence for India with 2.2 million cases, a 1.5-fold increase for East Asia region with 4.5 million cases, and a 1.6-fold elevation in prevalence for high‐income Asia‐Pacific and Southeast Asia regions with 183 and 199 thousand cases respectively.

**Conclusions:**

Our results showed an emerging epidemic for the prevalence of IBD in Asia regions and/or countries. Hence, we suggest the need for immediate action to control this increasing trend in Asia and Iran. However, we were virtually unable to use information about age groups, gender, and other factors influencing the evolution of IBD in our model due to lack of access to reliable data.

## Introduction

Inflammatory Bowel Disease (IBD) is a lifelong and nonfatal disease, or rather a global public health problem, marked by continuous cycles of remission and recurrence. In this respect, IBD poses multiple challenges for patients and the health care systems by undermining the quality of life of patients and imposing medication and care costs [1]. On the other hand, unlike some developed countries, we are still facing a marked increase in the incidence and/or prevalence of IBD in the Asian continent and developing countries such as Iran [2–4]. Hence, it is crucial to implement actions in the health settings to address the issue more effectively.

However, taking such actions need reliable and credible knowledge on the future epidemiology of the disease, which has not been well known despite multiple studies in Iran and Asia. Therefore, we must provide essential information to cope with this problem and design evidence-based planning. In other words, data on incidence, prevalence, mortality, and morbidity of IBD in the past, present and future are some essential components [5] for health policymakers to adopt clinical and management strategies [6] to make provision for IBD. Therefore, proper response to the burden of IBD requires obtaining accurate insight into the future prevalence of IBD.

In this regard, the projection studies are imperative to prepare health care systems for diseases. However, the selection of an appropriate method requires sufficient competence of the researcher as well as adequate epidemiological information to estimate the parameters of the model. For example, two previous studies in Canada [6] and Portugal [7] projected IBD prevalence using time series models. The use of these models requires information on the disease in several prior time units that are not available in Asia and Iran. Thus, we used an illness-death multi-state model to estimate the further prevalence of IBD in the Asian populations, Iran specifically, by 2035. The findings can be applied in the improvement of long-term management of IBD.

## Materials and methods

### Multi-state model

**Fig. 1:**
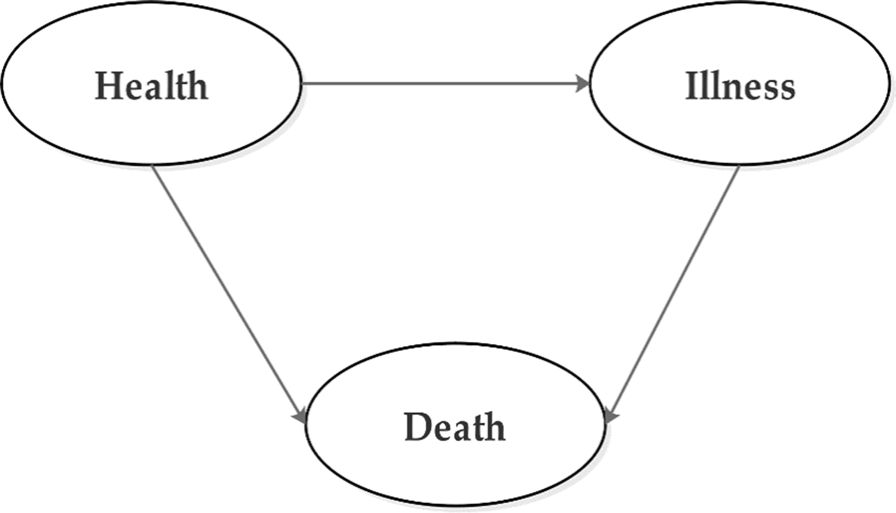
Illness death model

Multi-state models display a person's status over time for a particular process. The person has occupied a potential state at any given time but may also switch to other states [8]. Having evolved, these models have had numerous uses in epidemiology And have been widely used to model infectious [9] and chronic diseases [10]. The illness-death model (IDM) is a special multi-state model in which each person is put in a healthy, ill or dead condition, depending on the context, Fig. 1. The probabilities of transition between states include incidence rate, the mortality rate in healthy persons, and mortality rate in sick or IBD individuals. We did not consider the transition back to a healthy state because people with IBD do not usually recover.

### Model assumption

We first presumed that the incidence of IBD remained constant over time. This assumption is almost plausible for chronic conditions and has been observed in previous research [11, 12]. On the other hand, this assumption appears plausible because in stage three of the IBD evolutionary process, where Asian countries will be facing the issue in the not-too-distant future, we will see a rise in the prevalence even though the incidence is diminishing or even stable. Second, we assumed the mortality rate to be stable in healthy people and IBD patients. However, these values can be influenced by age, sex as well as time, which is inevitable in our research.

### Model inputs

**Fig. 2:**
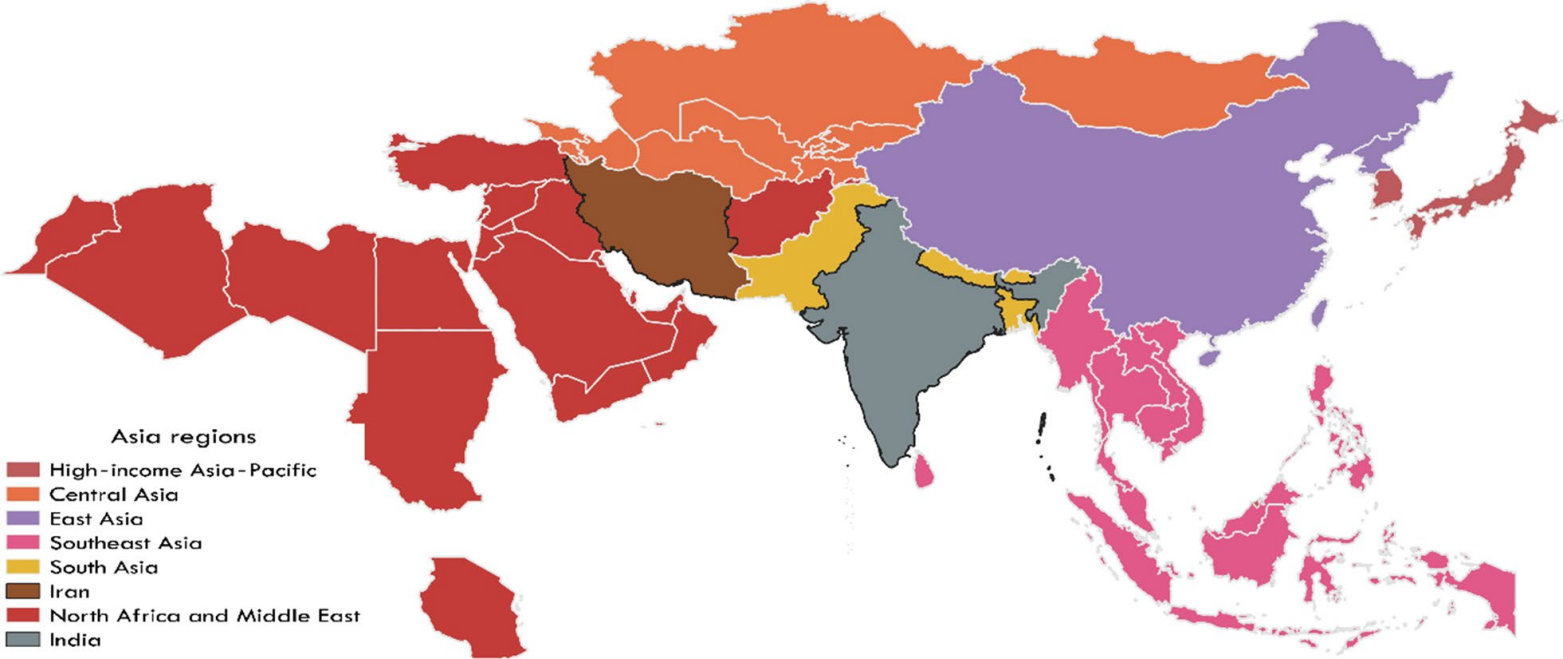
Asia region (country) map

Regarding the aforementioned section in this simulation study, we need information on the IBD incidence rate, the mortality rate in healthy individuals, and the mortality rate in IBD patients. Furthermore, we need data on the number of healthy persons, the number of sick people and the number of deaths at the starting point of the model, 2017, for each region and/or country. In this way, we first extracted the population size, fertility rate, number of new births and finally the death rate due to IBD and due to other diseases for regions of Asia, Iran particularly, from the Institute for Health Metrics and Evaluation (IHME) for the year 2017 [13, 14]. We also used two other pieces of evidence to extract the prevalence ratio and incidence rate of IBD for each region or country [15, 16]. Besides, we considered the reported IBD incidence rates in nine new articles according to the literature review [17–26] that were not included in the previous study [2]. We adjusted the geographical classification for incidence rates with the IHME classification as shown below:

**High‐income Asia‐Pacific region** including Brunei, Japan, Singapore and South Korea, Fig. 2. **Central Asia** including Armenia, Azerbaijan, Georgia, Kazakhstan, Kyrgyzstan, Mongolia, Tajikistan, Turkmenistan and Uzbekistan, Fig. 2. **East Asia** including China, North Korea and Taiwan, Fig. 2. **Southeast Asia** including Cambodia, Indonesia, Laos, Malaysia, Maldives, Mauritius, Myanmar, Philippines, Sri Lanka, Seychelles, Thailand, East Timor and Vietnam, Fig. 2. **North Africa and the Middle East** including Afghanistan**,** Algeria**,** Bahrain**,** Egypt**,** Iran**,** Iraq**,** Jordan**,** Kuwait**,** Lebanon**,** Libya**,** Morocco, Palestine**,** Oman**,** Qatar**,** Saudi Arabia**,** Sudan**,** Syria**,** Tunisia**,** Turkey**,** United Arab Emirates and Yemen, Fig. 2. **South Asia** including Bangladesh, Bhutan, India, Nepal and Pakistan, Fig. 2.

We did not include the countries of Israel and Cyprus in our estimates because in the IHME classification they have been classified in the Western Europe region. In addition, we did not consider the central Asia region because we could not find suitable information for the incidence rate of IBD in this region. We summarized the retrieved literature review data for Asia in Table1.

**Table 1:** Literature review-based data that were used to populate the projection models

Region(country)	Incidence rate	Prevalence(95% UI)	Death rate due to IBD (95% UI)	The death rate due to other causes(95% UI)
Iran	3.11	27.0 (24.6–29.6)	0.16 (0.06–0.19)	463.97 (452.58–477.04)
High‐income Asia‐Pacific	2.784	36.4 (33.7–39.3)	0.37 (0.3–0.49)	931.03 (921.89–940.28)
East Asia	1.541	134.6 (123.9–145.6)	0.34 (0.28–0.39)	752.33 (661.03–848.26)
Southeast Asia	0.804	15.3 (13.9–16.8)	0.22 (0.17–0.26)	651.29 (604–721.04)
North Africa and Middle East	3.121	29.6 (26.2–33.8)	0.17 (0.14–0.2)	509.71 (462.58–561.06)
South Asia (India)	8.133	16.2 (14.7–17.9)	0.3 (0.21–0.4)	675.31 (606.15–749.66)

### Model simulation

We used two stochastic and deterministic dynamic models to project IBD prevalence.

#### Deterministic approach

We first used a deterministic approach based on the ordinary differential equation to estimate the IBD prevalence as follow:

$$\begin{gathered} \frac{dHealth}{{dt}} = - (({\text{Death rate}}*Health) + ({\text{Incidence rate}}*Health)) + {\text{Birth number}} \hfill \\ \frac{dIBD}{{dt}} = ({\text{Incidence rate}}*Health) - ({\text{IBD death rate}}*IBD) \hfill \\ \end{gathered}$$

In this part, we used the desolve and FME packages of R to solve equations and also perform sensitivity analysis.

#### Stochastic approach

Because the real world is full of random phenomena, we used a stochastic model to estimate the future prevalent cases of IBD. For this purpose, we constructed a one-dimensional Markov matrix (3*3) compromised of healthy people, IBD patients and death transition probability as follows:

Then, a Mote-Carlo stochastic-based method was applied to solve the constructed matrix. We used ggplot2 R packages and QGIS3 to draw figures and maps respectively.

## Results

Given the markedly public health effect of IBD in Asia and subsequently in Iran, we designed this study to provide opportunities for policymakers to plan for IBD more efficiently.

### IBD's future trend in Iran

We observed an increasing trend in the prevalence of IBD from 2017 to now and from 2020 to 2035 in Iran, Fig. 2. Notably, it shifted from 23 thousand cases in 2017 to about 30 thousand cases in 2021 and it will be increased to about 69 thousand cases in 2035, Fig. 3 & Table2. We also estimated that the prevalence doubling time in Iran will be nearly 12 years, with 18,000 cases between 2020 and 2032. These findings show an ongoing IBD epidemic in Iran.

**Fig. 3:**
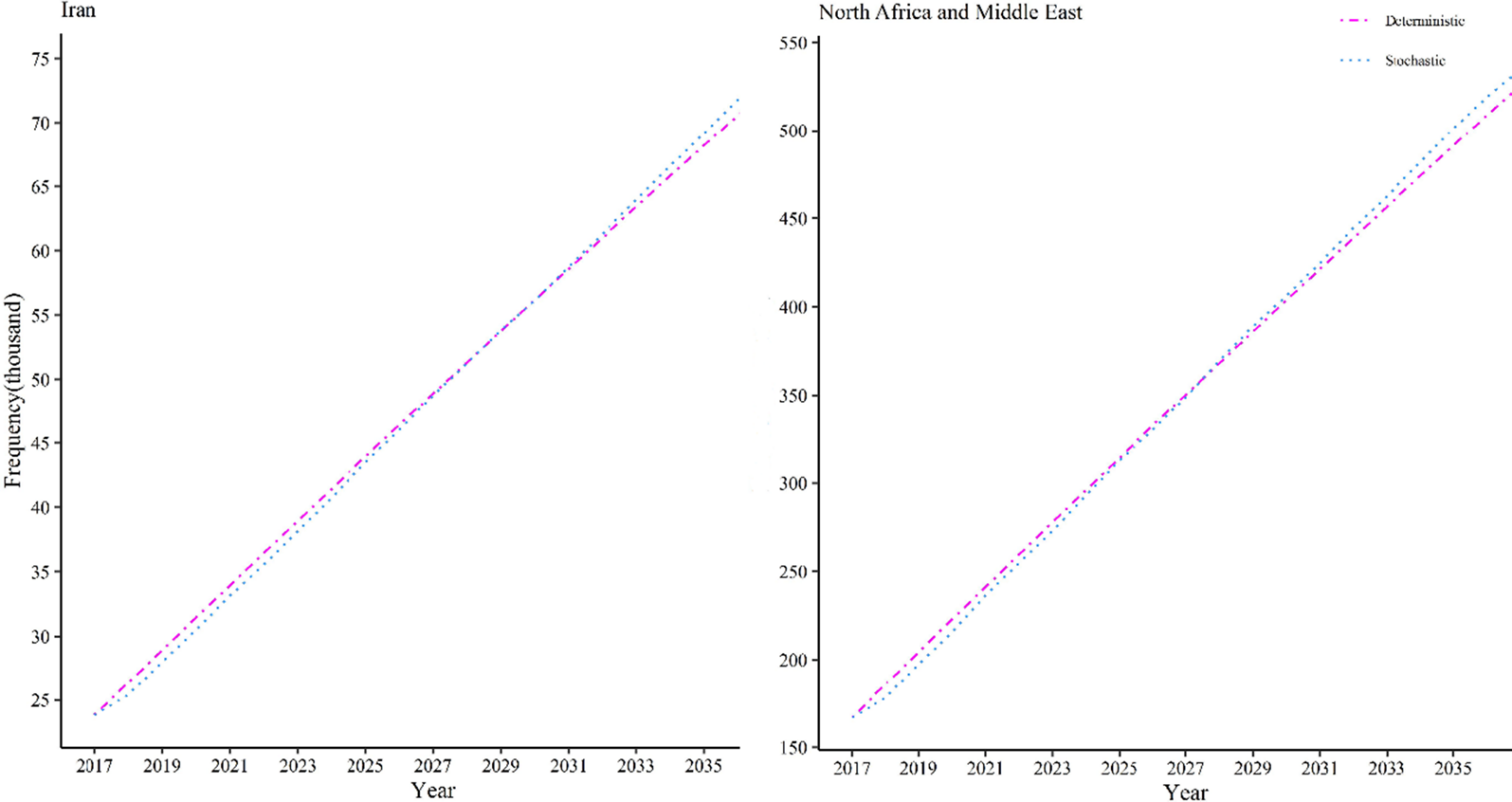
Prevalent IBD cases in Iran and North Africa and Middle East from 2017–2035

**Table 2:** Prevalent IBD cases in Asia region(country) from 2017–2035

Region (country)	2017	2020	2025	2030	2035
Iran					
Stochastic	23.812	30.475	43.505	56.177	69.166
Deterministic	23.812	31.445	43.955	56.207	68.207
High‐income Asia‐Pacific					
Stochastic	89.447	104.523	130.435	156.812	182.495
Deterministic	89.447	104.918	129.784	153.545	176.254
East Asia					
Stochastic	2.767	3.029	3.580	4.128	4.684
Deterministic	2.767	2.835	2.946	3.052	3.155
Southeast Asia					
Stochastic	103.884	117.154	142.512	169.552	199.35
Deterministic	103.884	119.716	145.481	170.490	194.766
North Africa and Middle East					
Stochastic	166.817	215.6	312.9	406.323	501.112
Deterministic	166.817	222.746	314.325	403.903	491.530
South Asia (India)					
Stochastic	212.451	550.156	1.115	1.685	2.244
Deterministic	212.451	549.418	1.097	1.627	2.142

### IBD's future trend in Asia

Likewise, we observed a growing trend in the prevalence of IBD for North Africa and the Middle East region, Fig. 2. Importantly, the prevalent IBD cases were shifted from about 166 thousand cases in 2017 to approximately 220 thousand cases in 2020 and will be expanded to nearly a half-million cases in 2035, Fig. 3 and Table2. We also observed an emerging prevalence for South Asia (India) and East Asia regions, Fig. 2. Hence, in India, the prevalence of IBD cases has changed from 212.451 thousand cases in 2017 to approximately 550 thousand cases in 2020 which will rise around 2.2 million cases in 2035, Fig. 4 and Table2. In the East Asia region, prevalent cases have increased from 2.767 million cases in 2017 to around 3 million cases in 2020 and it may increase to around 4.5 million cases in 2035, Fig. 4 and Table2. Similarly, we observed an emerging prevalence for High‐income Asia‐Pacific and Southeast Asia regions, Fig. 2. In High‐income Asia‐Pacific, the prevalence of IBD was shifted from 89.447 thousand cases in 2017 to around 105 thousand cases in 2020; it will be shifted to around 183 cases in 2035, Fig. 5 and Table2. In the Southeast Asia region, the IBD prevalent cases increased from 103.884 thousand cases in 2017 to approximately 118 thousand cases in 2020. The figure will be extended to around 199 thousand cases in 2035, Fig. 5 and Table2. From now on, this timespan for North Africa and the Middle East between 2031 and 2032 will be 425–444 thousand cases of IBD. In around 2025, India will experience the prevalence with 1.11 million cases. Prevalence doubling period for Southeast Asia, East Asia, and High‐income Asia‐Pacific regions were not observed in the 2035 period. These results indicate that the Asian continent will face a rapid acceleration in the prevalence of IBD by 2035. However, the rate of increase in the Asian continent is unbalanced.

**Fig. 4:**
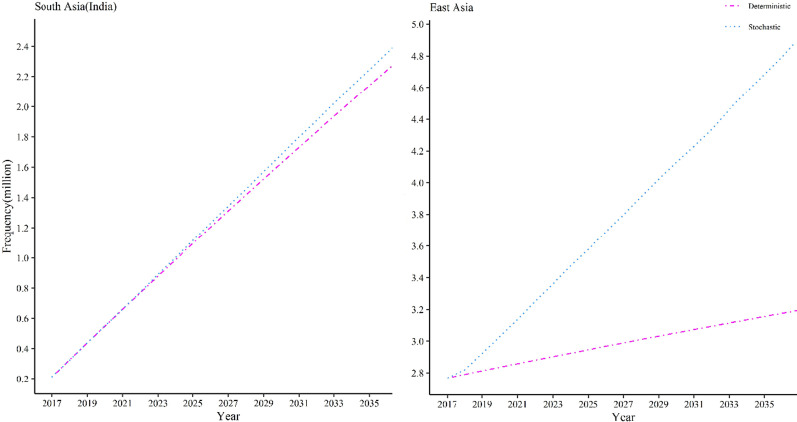
Prevalent IBD cases in South Asia and East Asia from 2017–2035

**Fig. 5:**
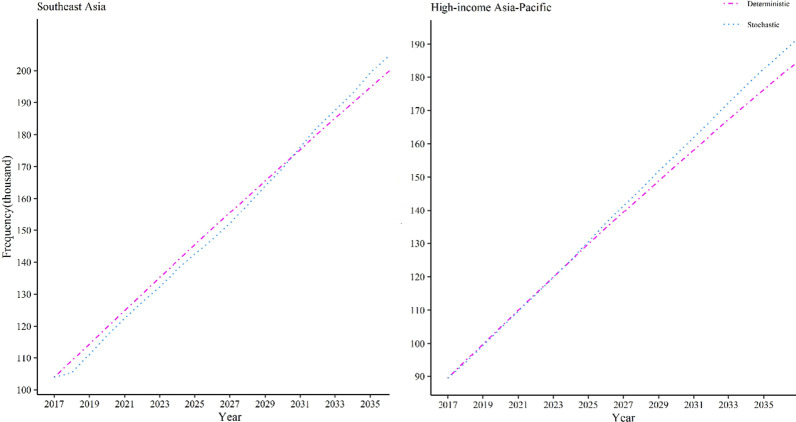
Prevalent IBD cases in Southeast Asia and High-income Asia–Pacific from 2017–2035

## Discussion

Our results pave the way to prepare health care systems for IBD. In other words, policymakers will have opportunities to take suitable measures and implement interventions. We observed an emerging epidemic of IBD in Asia and Iran by 2035. In Iran, we expect that the IBD prevalent cases will increase 2.5 times (fold) from 2021 until 2030. In North Africa and the Middle East, a 2.3-fold increment in prevalence is expected from 2020 to 2035. For India, the quadrupling of the prevalence from 2021 to 2035 was projected. In the East Asia region, a 1.6-fold increase in the number of prevalent cases was estimated by 2035. In High‐income Asia‐Pacific and Southeast Asia regions around 1.7 times elevation in prevalence was predicted by 2035. Our results emphasize the need for emergency action by the health policymakers in Asia and Iran to curb this increasing trend and subsequently emerging epidemic. According to previously eminent evidence [27], most of the countries included in our study and/or classified by IHME in Asia regions have just experienced two phases of IBD evolution(stages) till now, emergence and acceleration in incidence (shifting from sporadic cases to marked increase in the number of new cases). In other words, in Asia, we will confront two additional stages in the evolution of the IBD, compounding prevalence and prevalence equilibrium (increment and drop down in prevalent cases). But the pace of IBD evolution in some Asian regions tends to be higher than that in the Western world. As a result, the length of the struggle between incidence and death (stage 3) is expected to be shorter.

Similarly, the prevalence doubling period for Iran, North Africa and the Middle East, and India is much shorter than that reported in the Western world during 20 to 25 years [28, 29]. By contrast, it appears like we will not see Southeast Asia, East Asia, and High‐income Asia‐Pacific doubling in prevalence by 2035. In other words, the number of cases in these areas will not reach 235 thousand cases, around 6 million cases and 209 thousand cases, respectively. Hence, countries or regions of Asia are not homogeneous in terms of the epidemiological evolution of the disease. Apart from the capacity of the disease diagnostic system and the extent of access to healthcare, the obvious explanation may be related to the particular lifestyles of the inhabitants of those regions, i.e., the set of habits, relationships and beliefs [1]. The Asia–Pacific Crohn's and Colitis Epidemiology Study (http://www.access-apibd.com) is a good effort to address such differences and help understand IBD epidemiology. However, more multinational and multi-center studies seem necessary to explain the evolution of IBD in Asia more precisely.

In this milieu, there will be discrepancies in periodizing potential needs of the health system for disease management, which is beyond the scope of this article. However, in order to improve the management of IBD in Asian societies, and respond to this emergent epidemic, we need to tackle the issue from two perspectives, curbing the future incidence and provide affordable care for the future. From this standpoint, in the following lines, we attempt to clarify the strategies required by policymakers to provide an optimal platform for this adoption. We tried to provide a general concept with an emphasis on lifestyle theme. In this respect, Ashwin et al. [1] have made important efforts in their recent research. The researchers in this report, discussing multiple and established factors such as smoking, childbirth type, antibiotic usage, air quality and urban life in the development of IBD, made some general recommendations. Although the contribution of these variables to the evolution of IBD is small, virtually all of them are tied to a healthier lifestyle. On the other hand, in light of the epidemiological transition theory, preserving conventional dietary and behavioral patterns, i.e., locally appropriate inclusion of urbanized and westernized lifestyle is crucial to curb this emerged epidemic, because IBD is one of the modern lifestyle diseases. In 2016, modern lifestyle-related diseases were responsible for around 71% of all deaths [30]. In particular, sustainable development is a more rational alternative for globalization and/or modernization [31], where globalization and indices of sustainable development are interdependent [32]. We have sought to practice our workaround within the context of the Sustainable Development Goals (SDGs) [30]. However, it is unlikely to accomplish these goals without taking into consideration the underlying characteristics of nations, populations, and communities. Therefore, we suggest that policymakers design accurate and step-by-step field studies, attempt to include the imminent direction of modernity at the core of their culture to take cost-effective interventions. However, it should be remembered that these investigations are time intensive and expensive.

Inevitably, the prevalence of EBD in Asia will rise despite a decline in incidence. Therefore, to achieve sustainable health care and/or secondary prevention, Asian countries have serious future complications with respect to the number of cases, the provision of diagnostic and treatment facilities, access to health services, the longevity of the patient population, and the change of health care costs to biological agents [1]. Therefore, in general, it can be recommended that health authorities attempt to use more cost-effective interventions and resources.

The main drawback of our work was that we were practically unable to use partial differential equations (PDE) or other related approaches [11, 12, 33] to consider the effect of various age groups, gender and other influential factors in our model. This can lead to potential overestimation and/or underestimation in some areas. So that, age and gender differences were tangible in a related study in Canada [34]. However, we have not been able to use this information in the current model given the lack of access to suitable population-based information. The use of time-series models, on the other hand, often involves several years of disease information that was not present in our research. Another limitation of our research was that, despite an independent attempt for access to IBD incidence data in each region/country, the incidence of certain countries has been considered fixed for those countries since the publication of the last study. Lastly, in this report, we did not consider the impact of specific preventive approaches on our model. IBD does not have a known cause and the risk of different factors listed in the literature in its epidemiology is not entirely clear. However, despite the limitations mentioned above, this research is, as far as we know, the first attempt to clarify the epidemiological future of IBD in Asia and Iran. On the other hand, it should be recalled that the completion of IBD epidemiological puzzle studies, as well as population-based cohort and clinical trials, will take at least 10 to 15 years. Therefore, health authorities are strongly recommended to use the findings to more efficient planning and to prepare for the elevated prevalence.

## Conclusions

Our study indicates an increasing prevalence of IBD in Asia by 2035. As a result, health authorities need to make additional and optimized attempts at all levels of preventive initiatives, both primary and secondary, to curb the rising trend of IBD and provide sustainable health care. In this way, more resources are required for training, screening and recovery programs as well as evidence-based trials. However, given that IBD is a lifestyle disease, though, the collaboration among multiple management agencies of a community and not only health policymakers seem necessary.

## Data Availability

All the data used in this study is represented in Table 1.

## Abbreviations

IBD: Inflammatory bowel disease
UC: Ulcerative colitis
CD: Crohn's Disease
SP: Stochastic process
DP: Deterministic process
MM: Markove matrix
